# Sedentary Behavior in Childhood, Lower Arterial Compliance and Decreased Endothelial Function-Cross Sectional Data From a German School Cohort

**DOI:** 10.3389/fped.2021.787550

**Published:** 2022-02-17

**Authors:** Birgit Böhm, Hannah Kirchhuebel, Julia Elmenhorst, Jan Müller, Renate Oberhoffer-Fritz

**Affiliations:** Department of Sport and Health Sciences, Institute of Preventive Pediatrics, Technical University Munich, Munich, Germany

**Keywords:** endothelial (dys) function, arterial compliance, physical activity, physical fitness, children, sedentariness

## Abstract

**Background:**

Endothelial function by flow-mediated dilatation assesses early markers of atherosclerotic progression. Greater amounts of physical activity and physical fitness in children are associated with cardiovascular health benefits. We aimed to explore factors, influencing endothelial function and arterial compliance in a cohort of healthy school children.

**Methods:**

The 94 participants (41 girls, 53 boys) in the study were young, healthy children from a German school cohort. Anthropometric data, body composition and blood pressure were assessed. Blood was drawn (8 h overnight fast), assessing total cholesterol, high density lipoprotein and low density lipoprotein and triglycerides. Endothelial function was diagnosed by flow-mediated dilatation with ultrasonography (ALOKA/Hitachi, Prosound alpha 6). Tracking gates were set on the intima in B-mode. The waveform of diameter changes over the cardiac cycle was displayed in real time using the FMD-mode of the eTRACKING system. Changes in arterial diameter at baseline, ischaemia and vasodilatation were measured. A symptom limited pulmonary exercise test on a bicycle ergometer was performed to test cardiorespiratory fitness. Physical activity was assessed using GT3x accelerometers (Actigraph, USA), over 4 days (including 1 week-end day), with a minimum wear-time duration of 10 h.

**Results:**

The median age was 12.2 years (11.8–12.8). Children were normal weight, blood lipid profiles (cholesterol, high-density lipoprotein, low-density lipoprotein, triglyceride) were in normal range. Baseline measurements during the diagnostics of endothelial function revealed higher arterial compliance of the brachial artery in boys. Boys' cardiorespiratory fitness was higher than compared to girls. Boys met the recommendations of 60 min moderate to vigorous activity, whereas girls were significantly less active and did not meet current recommendations. More time spent in sedentary activity was the main predictor for lower arterial compliance (adjusted for age and sex), accounting for 14% of the variance. No significant model revealed, analyzing the influencing factors such as anthropometric data, blood lipids, physical activity and fitness on endothelial function.

**Conclusion:**

This is the first study on endothelial function in association to objectively measured physical activity and cardiorespiratory fitness in healthy school children in Germany. The study highlights the importance of reducing time spent being sedentary to maintain endothelial health.

## Introduction

Cardiovascular (CV) dysfunction, contributing to myocardial infarction, heart failure and stroke is one of the major cause of death in today's society worldwide (1). Increased arterial compliance and impairment of arterial endothelial function play an important role in the development of atheroclerosis and are strong predictors of CV events independent of traditional risk factors in adulthood (2, 3). Several pediatric studies have already reported reduced endothelial function (FMD) in children and adolescents at risk for atherosclerotic CV disease (4, 5).

High-frequency ultrasound is considered the gold standard for assessment of endothelial function in both adults (6, 7) and children (6).

Several studies have shown a relationship between cardiorespiratory fitness (CRF) and arterial compliance (8, 9). Veijalainen et al., for example, have given evidence that limited CRF is related to lower arterial compliance and higher arterial stiffness (10) measured by pulse wave velocity between carotid and femoral arteries. Equally important, it has been proven that physical activity may have a positive effect on reducing the blood pressure and the arterial stiffness in older adults (11, 12). It reduces the risk of cardiovascular diseases and has potential benefit on improved endothelial function (9).

Also, in obese children, it has been shown that the endothelial cell function can be improved significantly through a 12-week after school activity program. The school exercise intervention program leads to an improvement of vascular repair and endothelial cell function, leading toward an improved cardiovascular health (13). Similar relationships between exercise and vascular function in overweight children and adolescents are stated in the studies of Watts et al. and Woo et al. (14, 15).

The underlying mechanisms behind arterial remodeling seem to be complex. Factors such as shear stress, inflammation, sympathetic drive and oxidative stress lead to changes in cell signaling (16, 17). Especially the increased bioavailability of nitric oxide activated by signaling pathways after physical activity plays an important role in improving arterial compliance (18). The impact of exercise may be associated with the balance between reactive oxygen species (ROS) and the antioxidant defenses which influence the availability of nitric oxide (19).

Although the relationship between physical activity and arterial stiffness is complicated, the results of the studies mentioned above have given reasonable evidence regarding the positive effects of exercise training on arterial compliance. Reasoning based on these outcomes (20), reducing the cardiovascular risk factors may also be beneficial for children and prevent them from developing atherosclerosis.

However, studies investigating the associations between physical fitness, physical activity and arterial compliance and endothelial function in healthy children are limited. Currently no data in endothelial function in a German school children cohort exist.

Moreover, little is known of the effects of time spent being sedentary on arterial compliance and endothelial function in healthy children.

Therefore, our primary objective was to explore the factors influencing endothelial function, arterial compliance in a cohort of healthy school children and to quantify associations between physical fitness, physical activity, arterial compliance and endothelial function.

Our secondary objective was to examine possible sex differences on cardiorespiratory fitness and the amount of physical activity in different intensity levels in boys and girls in Germany. Girls are known to be less physically active than boys (21, 22) and spend less time in moderate to vigorous physical activity (23). A German cohort study further described lower physical fitness of girls compared to boys (24, 25). Earlier research of our study group demonstrated sex differences on A. carotid structure in healthy children (26). Further associations between physical activity intensity levels and arterial stiffness measured by pulse wave velocity have been described (27). It is, therefore, possible that endothelial function and arterial compliance may differ by childhood sex.

## Materials and Methods

### Study Design and Study Population

Data collection was part of the “Get fit-stay healthy” project funded by the German Heart Foundation, a prospective study conducted in Bavaria, Germany that already started in 2013. The study focused on cardiovascular risk screening in healthy children and adolescents with the main emphasis on arterial structure and function as well as endothelial function. Ethic approval was obtained from the Ethics Committee of the Faculty of Medicine, Technical University Munich (project number: 4027/11). All data were assessed prior to the Covid-19 pandemic, with lock down restrictions in Germany from March 2020 onwards. Data analysis took part between 2017 and 2019. All pupils (*n* =1 54) children from sixth grade of the participating school were asked to participate in the study. *N* = 105 volunteered to participate in the study. Written informed consent was given by the participating children and their guardians.

### Examination Process at the School

The study was performed under laboratory conditions in the medical room of the school. The room was quiet and temperature controlled. Temperature during diagnostics were 22°C (range 21–25°C, with a little ventilation if needed).

On the day of the examination the children were asked to come to school in a fasting state (8–10 h overnight fasting). The diagnostic routine started with the assessment of body composition, followed by blood collection and the diagnostic of endothelial function. The time of examination for body composition, blood collection and endothelial function was in the mornings between 7:30 and 11 am. After that the children had a light breakfast consistent of a banana and cereal, apple or orange juice and mineral water. The cardiovascular fitness test was performed later on the same day, in the same room with at least 1 h rest after breakfast. Finally, children were handed out the accelerometer which they wore for 7 days to objectively assess their physical activity.

### Assessment of Body Composition and Blood Pressure

Anthropometric measurements were assessed by trained staff according to standardized guidelines (28). Portable scales and stadiometers (Seca 799, MedicallLine, Hamburg, Germany) were used for quantifying body weight to the nearest 0.1 kg (in light sports clothing) and height to the nearest 0.1 cm. A non-flexible tape (Seca 201, Hamburg, Germany) was used for the measurement of waist circumference at the middle between last rib and the anterior superior iliac spine at the midclavicular line and of hip circumference at the anterior superior iliac spine. The body mass index (BMI) was calculated as weight in kilograms divided by height in meters-squared and converted into z-scores using the reference values of a German cohort (29). According to the German Obesity Association childhood overweight was defined as a BMI between 90^th^ and 97^th^ percentile, obesity was defined as a BMI greater than the 97^th^ percentile for children with the same age and sex. Underweight was defined as a BMI <10^th^ percentile (29).

Peripheral systolic and diastolic blood pressure were measured non-invasively at rest with a Mobil-O-Graph (IEM, Healthcare, Stolberg, Germany). Blood pressure measurements were performed on the left arm with the children in supine position. In order to select the appropriate arm cuff, subjects' arm circumferences were assessed before starting the tests.

Cuffs were chosen according to the measured left upper arm circumference (five different cuff sizes were used: 14–20 cm/20–24 cm/24–32 cm/32–38 cm/38–55 cm). The Mobil-O-Graph has already been validated for measurement of peripheral blood pressure (30, 31) and 24h ABPM (ambulatory blood pressure measurement) according to the BHS and ESH criteria (32, 33). Values were classified according to German age and sex specific norm values (20, 34).

### Laboratory Analysis

Blood was collected after at least an 8 h overnight fast in different tubes (Sarstedt, Nümbrecht, Germany) for preparation of serum or EDTA-plasma samples. All samples were processed within 2 h after sampling. If tests were not performed within the same day, the samples were stored frozen at −40°C. A complete blood count including reticulocytes was measured in EDTA-anticoagulated whole blood (Sysmex XE-5000). Total cholesterol, HDL-C, and triglycerides were assessed using routine methods on a Cobas Integra 800 analyzer (Roche Diagnostics, Mannheim, Germany). LDL-C was calculated according to Friedewald's formula (35).

### Measurements of Endothelial Function

Before initiating the diagnostic of endothelial function by flow-mediated dilatation (FMD) the examination was explained to the children. Children laid down in a supine position and rested for at least 10 min to guarantee stable conditions during measurement. The right arm was extended and immobilized with foam supports to guarantee a comfortable position. Since the A. brachialis is located medially, the arm is slightly turned outside (supination) to allow consistent imaging of the brachial artery.

Vascular endothelial function of the right brachial artery (BA) was studied with ultrasonography (Aloka/Hitachi, Prosound alpha 6) with a high-frequency (5–13 MHz) linear-array transducer. Mean arterial pressure was determined from the Mobil-O-Graph on the contralateral arm. The transducer was placed in the distal third of the upper arm to image the brachial artery. Tracking gates were set on the intima in B-mode, the artery resulting tracking lines, indicating the tracking position, are presented on the monitor in M-mode. The waveform of diameter changes over the cardiac cycle was displayed in real time using the FMD-mode of the eTRACKING system.

Changes in arterial diameter at baseline (1 min), ischaemia (5 min) and vasodilatation (2 min) were measured. Ischemia was developed by a blood pressure cuff placed around the forearm inflated to a pressure of 220 mmHg. After 5 min the cuff was deflated, causing increased flow-mediated vasodilatation. Peak artery diameter and the time taken to reach the maximal diameter after the release of the cuff, were recorded. From these data, FMD%, an index indicating the percentage dilated at the maximum vessel diameter in peak vasodilatation after cuff deflation, relative to maximum vessel diameter at baseline, was calculated. BA distensibility was defined by arterial compliance (AC), pressure strain elastic modulus (Ep), and PWV ß according to the following formula.

AC describes the ability of an artery to change its volume due to a given change in arterial blood pressure. The compliance is calculated from the diameter of the blood vessel (D) and BP.

AC = π(D_max2_-D_min2_)/[4(BP_max_-BP_min_)]

Pressure strain elastic modulus (Ep) is the ratio of stress and strain on the arterial wall and measures the intrinsic stiffness (Moo). An increase in stiffness leads to a higher Ep value.

Ep=(BP_max_-BP_min_)/[D_max_-D_min_)/D_min_]

Beta-Index (ß) is another parameter to depict arterial stiffness. The higher the ß-Index, the lower is the arterial elasticity.

B= ln(BP_max_/BP_min_)/[D_max_-D_min_)/D_min_]

PWV is the velocity of the pressure wave transmitted between two portions of the arterial tree (36). PWV β is measured as the local pulse wave velocity of BA, calculated from β.

PWV β = √((β^*^BP_min_)/(2p))

The echo-tracking system implemented in the ultrasound machine allows accurate measurements of diameter changes, based on radio frequencies (RF) signals, able to detect variations of the arterial diameters with a strictness of 0.01 mm (37).

### Assessment of Cardiorespiratory Fitness

Physical fitness was tested *via* a symptom limited pulmonary exercise test on a bicycle ergometer (Geratherm Respiratory, Ganshorn Medical, Germany). Baseline values were established during 2 min of rest followed by 2 min of unloaded pedaling. Afterwards, an increase in load was achieved *via* a ramp-wise protocol of 30 watts per minute. Criteria for ending the test was the maximum exhaustion of the subject and a drop of cadence below 60/min. The test featured a breath to breath gas exchange using a metabolic chart (Vmax Encore 229, SensorMedics, Viasys Healthcare, Yorba Linda, California). All exercise tests with a respiratory exchange ratio of >1.0 were discharged because of insufficient effort. Peak oxygen uptake was defined as the highest mean uptake of any 30 s time interval during exercise. Reference values for age, body mass, body height and sex, expressed in “% predicted” were calculated as previously described (38).

### Assessment of Physical Activity

Physical activity was assessed using GT3x accelerometers (Actigraph, USA), over 4 days (including 1 week-end day), with a minimum wear-time duration of 10 h. The accelerometer was positioned with an adjustable belt on the right iliac crest. Executive zeros over a period of 20 min were deleted. The time spent in inactivity as well as in light, moderate and vigorous PA was categorized using the cut-points per minutes (cpm) suggested by Evenson (39). Threshold counts < 100 cpm indicated physical inactivity, 101–2,295 cpm light PA, 2,296–4,011 cpm moderate PA and >4,012 cpm vigorous PA. Sedentary time was calculated as *wear time–(time spent in light PA* + *moderate PA* + *vigorous PA)* and total inactivity was defined as the sum of non-wear time and sedentary time. The time spent in different PA intensities was adapted for the number of days when the accelerometer was worn and expressed as minutes per day. All activity data measured by the ActiGraph GT3X+ was processed with the data analyzes software ActiLife, version 6.11.4 (ActiGraph, Pensacola, Florida, USA.

### Data Analysis

Statistical analyses were conducted using IBM SPSS Statistics (Version 23). The study cohort was characterized by standard descriptive statistics. Data for boys and girls were compared using the Mann-Whitney test. Data distribution was initially examined for normality using the Kolmogorov-Smirnov test. Due to non-normal distributed variables descriptive statistics were presented as median and interquartile range.

Due to significant differences in cardiorespiratory fitness and physical activity levels we calculated potential relations between anthropometric data, blood lipids physical fitness and activity and endothelial function separately for boys and girls. Univariate analysis between the variables was calculated with the Spearman's correlation coefficients. We further performed linear regression analysis to determine the main predictors for endothelial function and arterial compliance. Independent variables of the vascular function, were FMD (%, in systole) and arterial compliance (AC). FMD was adjusted for baseline diameter in systole, age and sex, whereas arterial compliance was adjusted for age and sex. As dependent variables BMI z-score, Hip to waist ratio, systolic and diastolic blood pressure z-scores, cholesterol, HDL-LDL ratio, daily physical activity, time spent in sedentary, light moderate and vigorous activity levels, VO_2max_ as well as Watt_max_ were integrated in the model as dependent variables. Criteria of normal distribution of the residuals were fulfilled so that logarithmic transformation was not necessary. Covariates were tested for collinearity. Correction for collinearity was not required as variance inflation factor (VIF) was <15. A value of *p* < 0.05 was considered to be statistically significant. *Post hoc*, compute achieved power was calculated; effect size *d* = 0.05; alpha error probability of 0.05; sample size of group 1 (*n* = 40) compared to sample size group 2 (*n* = 52). The outcome revealed the power (1-ß error prob = 0.745). Non-centrality parameter delta δ = 2.323, critical *t* = 1.662, Df = 85.85.

## Results

### Descriptive Characteristics in Boys and Girls

*N* = 105 participated in the study. Seven children were obese (*n* = 3 boys and *n* = 4 girls). Four boys demonstrated hypertensive blood pressure values. The data of these children were excluded from the analysis since they did not meet the criteria of being normal weight and normotensive. Overall, data of 94 healthy children (41 girls, 53 boys) were analyzed. Twelve children refused to have blood drawn. Two endothelial diagnostics had to be excluded due to artifacts during flow-mediated dilatation. All children did not take any medication, and did not suffer from known cardiovascular or metabolic disorder. The median age was 12.2 years (11.8–12.8). The characteristics of the sample population are displayed in Table 1.

**Table 1:** Characteristics of the study population.

	**Girls**			**Boys**						***p*-Values**
**Anthropometric data**	***n***	**Median**	**IQR**	***n***	**Median**	**IQR**	***n***	**Median**	**IQR**	
Age [years]	41	12.4	(11.9–12.8)	53	12.2	(11.8–12.9)	94	12.2	(11.8–12.9)	0.625
Height [cm]	41	156.0	(149.0–162.0)	53	155.0	(148.5–162.5)	94	156.0	(149.0–162.0)	0.994
Weight [kg]	41	44.1	(38.9–55.7)	53	45.0	(38.9–50.2)	94	44.6	(38.9–51.6)	0.661
BMI [kg/m^2^]	41	18.70	(16.05–22.25)	53	18.10	(16.15–19.95)	94	18.30	(16.10–20.80)	0.519
BMI z-score	41	0.08	(−1.11–1.17)	53	−0.17	(−0.81–0.64)	94	−0.820	(−0.854–0.730)	0.254
Hip circumference [cm]	41	76.0	(69.3–81.0)	53	74.0	(67.0–78.5)	94	75.5	(69.0–80.0)	0.153
Waist circumference [cm]	41	66.0	(60.3–74.0)	53	67.0	(62.0–72.8)	94	66.0	(62.0–73.8)	0.892
Hip to waist ratio	41	1.13	(1.08–1.16)	53	1.07	(1.05–1.12)	94	1.09	(1.05–1.14)	0.002^*^
**Blood lipids**										
Cholesterol [mg/dl]	31	175.00	(146.00–195.00)	41	166.00	(139.00–190.50)	72	167.00	(140.75–191.25)	0.400
HDL [mg/dl]	31	59.20	(46.80–76.30)	41	66.20	(57.45–76.40)	72	64.50	(53.10–76.30)	0.136
LDL [mg/dl]	31	95.30	(71.70–109.00)	41	88.60	(71.85–106.50)	72	91.50	(71.80–107.00)	0.336
Triglyceride [mg/dl]	31	57.40	(49.60–77.40)	41	47.30	(39.35–59.80)	72	53.35	(43.40–66.38)	0.015^*^
LDL_HDL_ratio	31	1.51	(1.14–2.24)	41	1.34	(1.05–1.73)	72	1.40	(1.08–1.87)	0.071
**Cardiovascular parameters and endothelial function**										
Heart rate [bpm]	40	79	(69–85)	52	73	(65–80)	92	74	(67–83)	0.017^*^
Ep [kPa]	40	307.03	(196.15–379.08)	52	249.78	(185.47–339.93)	92	276.25	(189.20–357.62)	0.127
ß-Index	40	27.54	(17.14–36.31)	52	22.45	(17.00–30.08)	92	23.98	(17.14–31.14)	0.119
Systolic blood pressure[mmHg]	40	108.00	(104.50–114.00)	52	110.50	(106.00–113.00)	92	109.50	(106.00–114.00)	0.761
Diastolic blood pressure [mmHg]	40	62.00	(56.25–67.00)	52	65.00	(58.50–68.00)	92	64.00	(57.25–68.00)	0.567
Systolic blood pressure z-score	40	0.05	(−0.21–0.27)	52	0.24	(−0.61–0.61)	92	0.108	(−0.275–0.367)	0.254
Diastolic blood pressure z-score	40	−0.46	(−1.37–0.15)	52	−0.15	(−1.15–0.44)	92	−0.302	(−1.293–0.44)	0.434
PWVß_BA [m/s]	40	10.41	(8.24–11.80)	52	9.46	(8.27–11.28)	92	10.03	(8.27–11.48)	0.234
Arterial compliance [mm^2^/kPa]	40	0.06	(0.04–0.08)	52	0.08	(0.04–0.10)	92	0.07	(0.04–0.10)	0.046^*^
Baseline diameter in systole [mm]	40	3.22	(2.94–3.84)	52	3.39	(3.01–3.98)	92	3.36	(2.98–3.94)	0.252
Peak diameter in systole [mm]	40	3.85	(3.36–4.67)	52	3.89	(3.47–4.75)	92	3.86	(3.43–4.67)	0.611
FMD_Systole [%]	40	10.72	(8.47–14.56)	52	12.47	(6.38–14.56)	92	11.23	(7.97–14.56)	0.631
Time to peak in systole[s]	40	59.00	(53.25–70.00)	52	55.00	(52.00–62.75)	92	58.00	(52.25–65.00)	0.969
**Physical activity and fitness**										
Sedentary activity [min/day]	41	337.57	(268.14–414.14)	53	330.14	(243.14–387.07)	94	333.29	(250.57–390.21)	0.556
Light activity [min/day]	41	148.00	(105.93–181.86)	53	151.07	(94.79–186.54)	94	150.71	(101.00–183.14)	0.739
Moderate activity [min/day]	41	42.57	(32.36–52.93)	53	57.07	(43.18–79.18)	94	49.00	(37.07–68.79)	0.005^*^
Vigorous activity [min/day]	41	3.14	(1.71–6.29)	53	4.64	(3.04–9.68)	94	4.00	(2.29–8.36)	0.032^*^
Very vigorous activity [min/day]	41	0.14	(0.00–0.43)	53	0.29	(0.04–1.57)	94	0.14	(0.00–0.93)	0.016^*^
MET rate	41	1.62	(1.49–1.77)	53	1.78	(1.57–1.98)	94	1.67	(1.51–1.90)	0.017^*^
Peak oxygen uptake [ml kg^−1^ min^−1^]	41	39.20	(33.30–41.75)	53	45.75	(40.48–51.50)	94	41.80	(37.25–47.15)	<0.001^**^
Peak workload [W]	41	145.0	(127.0–165.0)	53	165.5	(141.5–193.5)	94	152.0	(136.5–184.0)	0.011^*^
Maximal heart rate [bpm]	41	188	(182–196)	53	187	(180–195)	94	187	(181–196)	0.674

*Data are presented as median (interquartile range). Differences between boys and girls were analyzed by Mann Whitney U test*,

* *p < 0.05*;

** *p < 0.01*.

*BMI, body mass index; HDL, high density lipoprotein; LDL, low density lipoprotein; Ep, Pressure strain elastic modulus; ß-Index, ß stiffness index; PWVß, local pulse wave velocity measured at the brachial artery; FMD_systole, flow-mediated dilatation measured in systole; MET, metabolic equivalent of task*.

Boys and girls of the studies population did not differ in height, weight and BMI. Comparison of anthropometric differences between boys and girls only revealed a higher hip to waist ratio in girls 1.13 (1.08–1.16) compared to a 1.07 (1.05–1.17) (*p* = 0.002). Blood lipid profiles (cholesterol, high-density lipoprotein, low-density lipoprotein and triglyceride) were in normal range. Baseline measurements during the diagnostics of endothelial function revealed higher arterial compliance of the brachial artery in boys (*p* = 0.046) combined with a significantly lower heart rate at rest (*p* = 0.017), before cuff inflation. Boys also demonstrated higher VO2_max_ (*p* < 0.001) and Watt_max_ values (*p* = 0.011) during the cardiopulmonary exercise test than girls. All children reached their age and sex-specific norm values during the symptom limited pulmonary exercise test on a bicycle ergometer (38). There was no difference between maximum heart rate in boys compared to girls.

Regarding the physical activity level of the children, boys spent significantly more time doing moderate-intensity (*p* = 0.005), vigorous-intensity (*p* = 0.032) and very vigorous-intensity physical activity (*p* = 0.016), respectively. The median time spent in moderate- to vigorous-intensity physical activity was 61.71 min a day (IQR, 46.21–88.86) in boys, whereas the median time spent in moderate to vigorous activity of girls was only 45.71 min a day (IQR, 34.07–59.21).

No differences between the sex sub-groups existed in time spent being sedentary and the amount of time spent in light-intensity physical activity level. Overall, boys had a higher MET rate (*p* = 0.017).

### Relationships Between Anthropometric Data, Blood Lipids Physical Fitness, Physical Activity and Endothelial Function

Associations between anthropometric parameters, blood lipids physical fitness, physical activity, arterial compliance and endothelial function were analyzed for boys and girls as well as for the total study group. Results are displayed in Table 2.

**Table 2:** Relationships between anthropometric parameters, blood lipids, physical activity and fitness and arterial compliance (AC) and flow-mediated dilatation at time of systole (FMD_Systole_BA).

	**AC [l/kPa]**									**FMD_Systole_BA [%]**								
	**Girls**			**Boys**			**Total**			**Girls**			**Boys**			**Total**		
	***r***	***p***	***N***	***r***	***p***	***N***	***r***	***p***	***N***	***r***	***p***	***N***	***r***	***p***	***N***	**r**	***p***	***N***
**Anthropometric parameters**																		
Age [years]	0.016	0.923	40	0.181	0.200	52	0.119	0.257	92	0.047	0.774	40	0.024	0.868	52	0.024	0.823	92
Height [cm]	0.230	0.153	40	0.337^*^	0.015	52	0.329^**^	0.001	92	−0.051	0.753	40	0.037	0.794	52	−0.012	0.909	92
Weight [kg]	0.172	0.289	40	0.279^*^	0.045	52	0.240^*^	0.021	92	0.176	0.276	40	0.039	0.786	52	0.076	0.472	92
BMI [kg/m^2^]	0.151	0.351	40	0.066	0.640	52	0.081	0.442	92	0.284	0.076	40	0.022	0.88	52	0.120	0.255	92
Hip circumference [cm]	0.191	0.245	40	0.210	0.135	52	0.168	0.111	92	0.158	0.338	40	−0.098	0.488	52	0.007	0.948	92
Waist circumference [cm]	0.147	0.372	40	0.184	0.192	52	0.173	0.101	92	0.166	0.313	40	−0.053	0.71	52	0.036	0.733	92
Hip to waist ratio	0.018	0.911	40	0.092	0.515	52	−0.036	0.732	92	−0.036	0.827	40	−0.069	0.627	52	−0.004	0.973	92
Heart rate [bpm]	0.046	0.780	40	−0.009	0.951	52	−0.050	0.634	92	0.006	0.972	40	0.005	0.974	52	0.001	0.990	92
**Blood lipids**																		
Cholesterin [mg/dl]	−0.051	0.789	31	−0.078	0.632	41	−0.115	0.342	72	−0.003	0.988	31	−0.121	0.459	41	−0.071	0.559	72
HDL [mg/dl]	−0.121	0.525	31	−0.011	0.945	41	−0.030	0.807	72	−0.16	0.4	31	−0.031	0.853	41	−0.090	0.461	72
LDL [mg/dl]	−0.075	0.694	31	−0.028	0.864	41	−0.095	0.436	72	0.143	0.451	31	−0.246	0.131	41	−0.073	0.553	72
Triglyceride [mg/dl]	−0.006	0.977	31	0.023	0.886	41	−0.063	0.602	72	−0.017	0.929	31	0.13	0.424	41	0.086	0.481	72
LDL_HDL_ratio	0.082	0.667	31	−0.008	0.960	41	−0.034	0.78	72	0.158	0.404	31	−0.162	0.325	41	0.010	0.937	72
**Physical activity and fitness**																		
Daily activity [min]	−0.084	0.604	40	−0.169	0.231	52	−0.134	0.204	92	−0.083	0.611	40	0.074	0.6	52	0.028	0.790	92
Sedentary activity [min]	−0.222	0.168	40	−0.213	0.133	52	−0.235^*^	0.025	92	−0.241	0.135	40	0.221	0.118	52	0.032	0.762	92
Light activity [min]	0.058	0.721	40	−0.372^**^	0.007	52	−0.186	0.078	92	−0.256	0.111	40	0.047	0.744	52	−0.07	0.509	92
Moderate activity [min]	0.104	0.524	40	−0.142	0.320	52	−0.024	0.818	92	−0.097	0.551	40	−0.122	0.394	52	−0.105	0.320	92
Vigorous activity [min]	0.049	0.766	40	0.107	0.456	52	0.113	0.288	92	0.129	0.427	40	−0.153	0.285	52	−0.035	0.744	92
Very vigorous activity [min]	−0.101	0.536	40	0.092	0.521	52	0.079	0.454	92	−0.046	0.777	40	−0.084	0.558	52	−0.064	0.547	92
MET_rate	0.110	0.501	40	0.034	0.815	52	0.111	0.297	92	0.091	0.577	40	−0.259	0.067	52	−0.094	0.377	92
Peak oxygen uptake [ml/kg^−1^/min^−1^]	0.114	0.484	40	0.065	0.652	52	0.192	0.068	92	−0.086	0.598	40	−0.125	0.381	52	0.17	0.104	92
Peak workload [W]	0.348^*^	0.028	40	0.304^*^	0.030	52	0.380^**^	0.000	92	0.156	0.337	40	−0.058	0.685	52	0.358^**^	0.000	92
Maximal heart rate [bpm]	−0.166	0.306	40	0.105	0.467	52	−0.013	0.901	92	0.087	0.594	40	−0.304^*^	0.032	52	−0.04	0.713	92

*BMI, Body mass index; HDL, high density lipoprotein; LDL, low density lipoprotein; AC, arterial compliance; FMD_Systole_BA, Flow-mediated dilatation in systole measured at the A. Brachialis; MET, metabolic equivalent of task*.

* *p < 0.05*;

** *p < 0.01*.

In boys, height was positively associated to AC (*r* = 0.337, *p* = 0.015), and weight (*r* = 0.279, *p* = 0.045) demonstrated a positive relation to AC. Better physical fitness, especially the maximal workload was positively correlated to AC (*r* = 0.304, *p* = 0.030). Regarding the amount physical activity, more time spent in light activity was negatively correlated to AC (*r* = −0.372, *p* = 0.007). A negative correlation was observed between FMD% and the maximum heart rate in boys (*r* = −0.304, *p* = 0.032).

An indicator for physical strengths and fitness is the performance measured in Watt during the cardiorespiratory fitness test. In girls a positive correlation was observed between the maximum workload (Watt) and AC (*r* = 0.348, *p* = 0.028).

No associations revealed between parameters of arterial stiffness and endothelial function with the blood lipid profiles.

### Predictors for Arterial Compliance and Endothelial Function

Next to the calculation of associations, we further assessed the main influencing factors for arterial compliance in children. After adjustment for age and sex (model 1), the linear regression analysis revealed that more time spent in sedentary activity (ß = −0.280, *p* = 0.037) was the main predictor for lower arterial compliance, accounting for 14% of the variance [*R*^2^ = 0.014, *F*_(13,57)_ = 0.767]. Taking BMI and systolic blood pressure also into account the model still revealed significant (*R*^2^ = 0.0122, *F*_(10,60)_ = 0.744, *p* = 0.027) (Table 3).

**Table 3:** Multivariable correlates of arterial compliance.

	**Model 1 AC**				**Model 2 AC**			
	**ß**	**SE**	***P***	**95.0% CI**	**ß**	**SE**	***P***	**95.0% CI**
BMI (z-score)	0.253	0.253	0.260	[−0.184, 0.667]	-	-	-	
Hip to waist ratio	0.082	0.082	0.559	[−3.375, 6.174]	0.076	2.333	0.570	[−3,194,5.743]
Systolic blood pressure (z-score)	−0.159	−0.159	0.302	[−0.780, 0.246]	-	-	-	
Diastolic blood pressure (z-score)	0.114	0.114	0.459	[−0.212, 0.464]	0.048	0.149	0.728	[−0.246, 0.350]
Cholesterol [mmol/dl]	0.125	0.125	0.478	[−0.008, 0.017]	0.064	0.006	0.701	[−0.009, 0.014]
LDL/HDL ratio	−0.116	−0.116	0.500	[−0.871, 0.430]	−0.047	0.297	0.767	[−0.682, 0.506]
Daily physical activity [min/day]	0.052	0.052	0.697	[−0.009, 0.013]	0.081	0.005	0.538	[−0.007, 0.014]
Sedentary activity [min/day]	−0.280	−0.280	0.037	[−0.001, 0.000]	−0.295	0.000	0.027	[−0.001, 0.000]
Light activity [min/day]	−0.047	−0.047	0.776	[−0.001, 0.001]	−0.092	0.000	0.569	[−0.001, 0.001]
Moderate activity [min/day]	0.004	0.004	0.988	[−0.003, 0.003]	0.059	0.001	0.791	[−0.002, 0.003]
Vigorous activity [min/day]	−0.023	−0.023	0.901	[−0.010, 0.009]	−0.027	0.005	0.886	[−0.010, 0.009]
Peak oxygen uptake [ml/kg^−1^/min^−1^]	0.107	0.107	0.625	[−0.042, 0.069]	−0.024	0.021	0.885	[−0.044, 0.038]
Peak workload [W]	0.073	0.073	0.701	[−0.009, 0.013]	0.165	0.005	0.320	[−0.005, 0.014]

*ß, Standardized coefficient beta; CI, Confidence interval; BMI, Body mass index; LDL, low density lipoprotein; HDL, high density lipoprotein*.

*Model 1: AC, Arterial compliance adjusted for age and sex as corvariates; n = 70, R^2^ = 0.014, F_(13,57)_ = 0.767, p = 0.037*.

*Model 2: AC, Arterial compliance adjusted for age, sex, BMI and systolic blood pressure as covariates; n = 70, R^2^ = 0.0122, F_(10,60)_ = 0.744, p = 0.027*.

To determine the influencing factors for endothelial function in the total study group, FMD % in systole was adjusted for baseline diameter, age and sex (model 1) *R*^2^ = 0.098, *F*_(13,57)_ = 0.479, *p* = 0.928 and further also adjusted for BMI and systolic blood pressure. The regression also did not analysis reveal a significant prediction model *R*^2^ = 0.077, *F*_(11,59)_ = 0.449, *p* = 0.926 (Table 4).

**Table 4:** Multivariable correlates of endothelial function measured by flow-mediated dilatation.

	**Model 1 FMDsys [%]**				**Model 2 FMDsys [%]**			
	**ß**	**SE**	***P***	**95.0% CI**	**ß**	**SE**	***P***	**95.0% CI**
BMI (z-score)	0.122	0.213	0.595	[−0.312, 0.540]	-	-	-	
Hip to Waist ratio	0.125	2.386	0.389	[−2.708, 6.849]	0.094	2.211	0.490	[−2.888, 5.960]
Systolic blood pressure (z-score)	0.097	0.256	0.541	[−0.356, 0.671]	-	-	-	
Diastolic blood pressure (z-score)	0.074	0.169	0.640	[−0.259, 0.418]	0.088	0.147	0.532	[−202, 0.388]
Cholesterol [mmol/dl]	−0.044	0.006	0.810	[−0.014, 0.011]	−0.070	0.006	0.682	[−0.014, 0.009]
LDL/HDL ratio	−0.078	0.325	0.661	[−0.794, 0.507]	−0.051	0.294	0.756	[−0.680, 0.496]
Daily physical activity [min/day]	−0.110	0.006	0.430	[−0.016, 0.007]	−0.115	0.005	0.396	[−0.015, 0.006]
Sedentary activity [min/day]	−0.195	0.000	0.155	[−0.001, 0.000]	−0.210	0.000	0.119	[−0.001, 0.000]
Light activity [min/day]	−0.054	0.000	0.753	[−0.001, 001]	−0.055	0.000	0.742	[−0.001, 0.001]
Moderate activity [min/day]	−0.184	0.001	0.434	[−0.004, 0.002]	−0.169	0.001	0.458	[−0.004, 0.002]
Vigorous activity [min/day]	0.113	0.005	0.557	[−0.007, 0.012]	0.560	0.005	0.578	[−0.007, 0.012]
Peak oxygen uptake [ml/kg^−1^/min^−1^]	−0.011	0.028	0.962	[−0.057, 0.054]	−0.058	0.020	0.734	[−0.048, 0.034]
Peak workload [W]	−0.005	0.006	0.982	[−0.011, 0.011]	0.007	0.005	0.967	[−0.009, 0.010]

*ß, Standardized coefficient beta; CI, Confidence interval, FMD_sys_ [%], flow-mediated dilatation in systole adjusted for age, sex and baseline diameter at systole; BMI, Body mass index; LDL, low density lipoprotein; HDL, high density lipoprotein*.

*Model 1: FMDsys, Flow-mediated dilatation adjusted for baseline diameter at systole, age and sex as corvariates; n = 70, R^2^ = 0.098, F_(13,57)_ = 0.479, p = 0.928*.

*Model 2: FMDsys, Flow-mediated dilatation adjusted for baseline diameter at systole, age sex, systolic blood pressure and BMI as covariates; n = 70, R^2^ = 0.077, F_(11,59)_ = 0.449, p = 0.926*.

## Discussion

The purpose of this study was to assess factors influencing the arterial compliance and endothelial function in healthy children, taking possible sex differences into account. Our data yielded two important findings:

Firstly, after adjustment for age and sex, the main predictor the amount of time spent in sedentary behavior was the main predictor for lower arterial compliance. Secondly, sex differences exist regarding physical activity levels, cardiorespiratory fitness and arterial compliance.

### The Impact of Physical Activity on Arterial Compliance and Endothelial Function

Sedentary lifestyle increases the risk of prematurely developing of atherosclerosis. Regular physical activity affects endothelial function (40). The results of our study emphasize the importance of reducing and limiting the amount of time spent being sedentary, particularly the amount of recreational screen time which is recommended in the current guidelines of the World Health Organization (41). Physical activity can be described by duration of the activity and level of intensity (light, moderate, vigorous and very vigorous activity). Abbott et al. (42) described a significant relationship between physical activity and FMD%, even among a group of moderately active children. This gives credence to the importance of physical activity in early childhood. However, the underlying mechanisms behind arterial remodeling are complex. Training induces a direct impact on the vasculature (43). The pattern of blood flow and the amount of shear stress that occur during exercise may be related to the specific training characteristics, including training intensities (44). Next to moderate training intensity, high-intensity interval training is relationed to vascular function for improving the range of physiological, functional and clinical parameters, including endothelial function (45).

Children's general play and physical activity is characterized by changes in intensity levels and repeated sprint running is a natural exercise for children. A classical analysis reported that children have a preference for intermittent, explosive and intense activities of very short duration (< 15 s), which causes partly anaerobic-lactic metabolic conditions (46).

In the present study, girl's physical activity was lower than in boys. Further our data demonstrated that time spent in vigorous and very vigorous activities was significantly lower in girls than in boys. Girls only spent 45.71 min a day (IQR, 34.07–59.21) in moderate to vigorous activity and did not meet the recommendations of 60 min per day, which would confer to multiple beneficial health outcomes, such as cardiorespiratory fitness (47) cardiometabolic health (48) and cardiovascular health (49).

There is a need to reduce sedentary time and especially motivate girls to more intensive activities or to design sex-specific interventions with special attention to vigorous and very vigorous physical activity intensities in girls (e.g., ball games, robe skipping, running activities).

Favorite sports in boys (e.g., ball games and team sports such as soccer and basketball) can be defined as activities and sports with changing intensity levels similar to interval training (50).

Lower levels of physical activity have been related to stiffer arteries measured by pulse wave velocity between the femoral artery and low vascular function (51, 52).

Our findings are further in line with Veijalainen et al. (10) who observed that children with less stiff arteries have higher levels of unstructured physical activity and cardiorespiratory fitness. This was especially the case in boys whereas girls were in the opposite halve of these variables (10). However, though the authors found no associations between time spent in sedentary behavior and arterial stiffness (10), we found that time in sedentary behavior was the main predictor of lower arterial compliance, describing 14% of the evidence.

In the present study we could not demonstrate relationships between FMD and fitness after adjustment for age and sex. It has to be kept in mind that in the present population the most established risk factors for endothelial dysfunction such as smoking or hypercholesterolaemia are lacking (4). Previous studies reported data on the beneficial effect of exercise on endothelial health are most consistent in subjects with impaired endothelial function (53). One of the suggested new training types could be aerobic interval training, since data indicate that this type of training is superior in reversing endothelial dysfuction in children (54, 55).

Endothelial maximum dilatation is primarily due to endothelial cells releasing relaxing factors. To find out whether the FMD is solely dependent on the endothelium, a vasodilation through the direct action of the smooth muscles must be investigated. In adults, this is achieved by measuring vasodilation in response to glyceryl trinitrate (GTN) (42). However, it was ethically not possible to administer GTN to young healthy children in this voluntary prevention study.

In this context the time to peak is an indicator for endothelial health. Data in the present study showed that the time to peak dilation was 58 s. (sys, median) and the IQ range was 52.25–65.00 s. Our results are in line with results of Hopkins et al. who described a mean time of 60.7 (95%CI 59.0–62.5) s. to peak dilatation (56). The results are further comparable to the data of Järvisalo et al. They reported the highest mean dilation at 70.00 s. after cuff release in healthy children and adolescents (9–16 years). The majority of the subjects obtained their dilation peak diameter between 40 and 120 s, presenting a large range in response. Järvisalo et al. concluded that for true FMD peak response brachial artery diameter measurement is necessary up to 120 s. after cuff release as there is a certain variety in healthy children and adolescents (57).

Measurement of endothelial function by FMD is non-invasive, reproducible and non-painful (6), so it is particularly suitable for the investigation of young adults and children with the earliest stages of atherosclerosis, hence, providing the best opportunity for prevention (7).

Our data showed no sex differences in baseline diameter and also endothelial function, assessed by FMD was similar in both sexes. Our finding goes in line with Pahkala et al. (58). The group studied over 500 healthy boys and girls (13 years old) and reported no significant difference in endothelial function (FMD). In contrast, Hopkins et al. (56) described significant difference between boys and girls.

In this study we focused on an apparently healthy sample of school children.

Currently no reference values have been published for endothelial function assessed by FMD in children in a German cohort.

### Strengths and Limitations of the Study

The major strengths of the study are that we used well established diagnostic measures for the assessment of physical examination, laboratory, ultrasound data as well as objective measurement of physical activity and cardiorespiratory fitness.

We also used established ultrasound diagnostics to assess the flow-mediated dilatation of the brachial artery as a response to a physiological stimulus (shear stress). Since we performed our diagnostics in healthy children we did not measure smooth muscle dependent vasodilatation as a response to exogenous sources of NO such as nitroglycerin due to ethical considerations. Another limitation of the study is that we did not have data on the puberty status of the children. From the possible *n* = 154 pupils only 68% volunteered to participate in the study. This might be due to the fact that the examinations took place in the mornings during school hours.

## Conclusion

This is the first study on endothelial function in association to objectively measured physical activity and cardiorespiratory fitness in healthy school children in Germany.

Time spent in sedentary behavior was the main predictor for lower arterial compliance in healthy children. Girls were less active than boys and did not meet current WHO guidelines for moderate-intensity activity.

This highlights the importance of maintaining or making every effort to promote physical activity to an average of 60 min per day per week and to reduce time being sedentary in childhood (41). Among the lifestyle-related factors measured in the present study, time spent in sedentary behavior has a stronger effect than BMI or blood lipids or cardiovascular fitness, on lower arterial compliance in healthy children.

Future studies should consider behavior approaches to further investigate differences and preferences of physical activity in boys and girls, resulting in possible sex-specific activity interventions. Prospective studies should further deepen the knowledge of vascular adaptations with regard to different activity intensities in healthy children, resulting in a better understanding of the promotion of an active healthy lifestyle to prevent cardiovascular risk factors and endothelial dysfunction.

The promotion of an active and healthy lifestyle becomes even more important, considering the fact that the present data was collected before the corona pandemic. Due to an increased sedentary behavior and an increased lack of exercise during the pandemic, even more far-reaching effects on the health of children must be feared.

## Data Availability Statement

The raw data supporting the conclusions of this article will be made available by the authors, without undue reservation.

## Ethics Statement

The studies involving human participants were reviewed and approved by Ethics Committee of the Faculty of Medicine, Technical University of Munich (project number: 4027/11). Written informed consent to participate in this study was provided by the participants' legal guardian.

## Author Contributions

RO-F, BB, JE, and JM contributed to the conception and design of the study. BB was main responsible for data collection as project coordinator and drafted the manuscript. BB, JM, and JE were involved in the practical work. BB, JE, JM, and HK contributed to the statistical analysis and interpretation of the data. HK assisted drafting the manuscript and literature research. RO-F, JM, and HK critically revised the manuscript. All gave final approval and agreed to submit the work.

## Funding

The study was funded by the German Heart Foundation within the project Get fit-Stay healthy–Cardiovascular risk screening in healthy German children in Bavaria. Publication of this open access article was supported by the German Research Foundation (DFG) for University libraries.

## Conflict of Interest

The authors declare that the research was conducted in the absence of any commercial or financial relationships that could be construed as a potential conflict of interest.

## Publisher's Note

All claims expressed in this article are solely those of the authors and do not necessarily represent those of their affiliated organizations, or those of the publisher, the editors and the reviewers. Any product that may be evaluated in this article, or claim that may be made by its manufacturer, is not guaranteed or endorsed by the publisher.
